# Involvement of JMJ15 in the dynamic change of genome-wide H3K4me3 in response to salt stress

**DOI:** 10.3389/fpls.2022.1009723

**Published:** 2022-09-26

**Authors:** Yuan Shen, Yuhao Chi, Shun Lu, Huijuan Lu, Lei Shi

**Affiliations:** ^1^School of Pharmacy, Xinxiang Medical University, Xinxiang, China; ^2^School of Basic Medical Sciences, Xinxiang Medical University, Xinxiang, China

**Keywords:** jumonji demethylase, H3K4me3, JMJ15, WRKY46, WRKY70, salt stress

## Abstract

Post-translational histone modifications play important roles in regulating chromatin structure and transcriptional regulation. Histone 3 lysine 4 trimethylation (H3K4me3) is a prominent histone modification mainly associated with gene activation. Here we showed that a histone demethylase, JMJ15, belonging to KDM5/JARID group, is involved in salt stress response in *Arabidopsis thaliana*. *Jmj15* loss-of-function mutants displayed increased sensitivity to salt stress. Moreover, knockout of *JMJ15* impaired the salt responsive gene expression program and affected H3K4me3 levels of many stress-related genes under salt-stressed condition. Importantly, we demonstrated that *JMJ15* regulated the expression level of two WRKY transcription factors, *WRKY46* and *WRKY70*, which were negatively involved in abiotic stress tolerance. Furthermore, JMJ15 directly bound to and demethylated H3K4me3 mark in the promoter and coding regions of *WRKY46* and *WRKY70*, thereby repressing these two *WRKY* gene expression under salt stress. Overall, our study revealed a novel molecular function of the histone demethylase JMJ15 under salt stress in plants.

## Introduction

Higher plants can adapt to changing environmental conditions in different ways via adjustments in gene expression. Epigenetic regulation of gene expression involving chromatin modification such as histone 3 lysine 4 trimethylation (H3K4me3) plays essential roles in plant response to environmental conditions (van Dijk et al., 2010; Foroozani et al., 2021). H3K4me3 has long been known as an active mark for gene transcription (Santos-Rosa et al., 2002; Li et al., 2007). Genome-wide analysis revealed that a large number of genes were marked by H3K4me3 which predominantly found at the promoter and 5′ end of genes in plants (Zhang et al., 2009). H3K4me3 was often associated with stress-induced gene expression by plant internal and external signals (Sokol et al., 2007; Kim et al., 2008; van Dijk et al., 2010), suggesting an active transcription role for H3K4me3. However, in some cases the increase of H3K4me3 was found to be lagged behind stressed gene activation (Kim et al., 2008; Hu et al., 2011), indicating that H3K4me3 might have a function to mark active gene status. In addition, H3K4me3 has also been found to play a role in transcriptional memory of stress-responsive genes in plants (Jaskiewicz et al., 2011; Ding et al., 2012; Kim et al., 2012, 2015). H3K4 methylation is mediated by the Trithorax group proteins (TRX), including five Trithorax-like (ATX) proteins and two ATX-related (ATXR) proteins in *Arabidopsis* (Ng et al., 2007). *Arabidopsis* ATX1, ATX4, and ATX5 were found to be necessary for abscisic acid (ABA) and dehydration stress responses (Ding et al., 2011; Liu et al., 2018).

Histone methylation could be reversed by histone demethylases. Lysine Specific Demethylase 1 (LSD1) was the first identified histone demethylase to remove mono- and di-methyl groups from H3K4 (Shi et al., 2004). Jumonji C (jmjC) domain-containing proteins were the second known class of histone demethylases. JmjC proteins, conserved in plants, animals and fungi, catalyze histone lysine demethylation through a ferrous ion [Fe(II)] and a-ketoglutaric acid (a-KG)-dependent oxidative reaction (Tsukada et al., 2006). JmjC domain containing demethylases are divided into distinct groups including KDM2A/JHDM1A, KDM5/JARID, KDM4/JMJD2, KDM6/JMJD3, and KDM3/JMJD1/JHDM2, depending on their binding and catalytic specificities. In humans, the KDM5/JARID group demethylases specially catalyze H3K4me2/3 demethylation (Mosammaparast and Shi, 2010). The *Arabidopsis* genome encodes ∼20 jmjC domain-containing histone demethylases (JMJ11–JMJ31), among which six jmjC proteins (JMJ14, JMJ15, JMJ16, JMJ17, JMJ18, and JMJ19) belonging to KDM5/JARID group demethylate H3K4me1/2/3 (Lu et al., 2008; Sun and Zhou, 2008; Chen et al., 2011). JMJ14 demethylates H3K4me3 *in vitro* and represses floral integrator genes Flowering Locus T (*FT*) and Suppressor of overexpression of CO1 (*SOC1*) by removing H3K4me3 (Jeong et al., 2009; Lu et al., 2010; Yang et al., 2010, 2018). JMJ16 negatively regulates leaf senescence through repressing the positive regulators of *WRKY53* and *SAG201* by reducing H3K4me3 (Liu et al., 2019). JMJ15 and JMJ18 regulate flowering time by demethylating H3K4me3 at the locus of the floral repressor gene Flowering Locus C (*FLC*) (Yang et al., 2012a,b). In addition to the essential roles in plant development, JMJ15 and JMJ17 are also involved in stress response. The *jmj17* loss-of-function mutants display dehydration stress tolerance and ABA hypersensitivity (Huang et al., 2019; Wang et al., 2021). In detail, JMJ17 regulates the stomatal closure and ABA response through modulating *OPEN STOMATA1* (*OST1*) and *ABI5* genes by demethylating H3K4me3 (Huang et al., 2019; Wang et al., 2021). Our previous results showed that JMJ15 participated in salinity stress response (Shen et al., 2014). *Jmj15* gain-of-function mutants increased tolerance to salinity stress and down-regulated many genes encoding transcription regulators involved in stress responses (Shen et al., 2014). Although the overexpression of *JMJ15* confers tolerance to salt stress, the molecular mechanism of JMJ15 mediated salt tolerance remains elusive.

In the present study, we showed that two *jmj15* loss-of-function mutant alleles (*jmj15-3* and *jmj15-4*) revealed more sensitive phenotypes to salinity stress. Importantly, the *jmj15* loss-of-function mutations led to up-regulation of many stress-related genes under salt condition rather than under normal condition. In addition, chromatin immunoprecipitation sequencing (ChIP-seq) showed that knockout of *JMJ15* regulated H3K4me3 levels of many salt and water deprivation stress-related genes under salt-stressed condition. Furthermore, our results showed that JMJ15 directly bound to and repressed the genes of *WRKY46* and *WRKY70* that played negative roles in salt stress tolerance. Taken together, our data unravel a novel molecular function of JMJ15 in salt stress responsiveness, which is distinct from the function of other characterized jmjC genes in *Arabidopsis*.

## Materials and methods

### Plant materials and growth conditions

In this study, *Arabidopsis thaliana* wild type Col-0 and mutant lines *jmj15-1* (GABI_257F10), *jmj15-2* (GABI_876B01), *jmj15-3* (GABI-663C11), and *jmj15-4* (GABI-229F03) in the Col-0 background were used. The insertional knock-out lines *jmj15-3* (Shen et al., 2014) and *jmj15-4* (Yang et al., 2012b) have been previously characterized. The gain-of-function T-DNA mutant lines *jmj15-1*, j*mj15-2*, and the over-expressing tagged line *35s-JMJ15-HA* have been reported (Shen et al., 2014). The *Arabidopsis* seeds were surface-sterilized by 5% (w/v) sodium hypochlorite for 7 min, washed with 95% (v/v) ethanol twice, and then sown on 1/2 Murashige and Skoog (MS) medium. After stratification in darkness at 4°C for 2 days, seeds were transferred into a growth chamber (20°C) under white light (120 μmol m^–2^ s^–1^) in 16 h light photoperiods. Wild-type and mutant plants were grown together and their mature seeds were harvested at the same time to avoid differences in post-maturation that can affect seed germination. Seeds of each genotype were harvested as a single bulk consisted of at least five plants. Seeds were stored in open tubes inside a closed box and maintained in darkness with silica gel at 4°C until the experiments were performed.

### Seed germination assay

For measuring seed germination, more than 100 seeds of each genotype (Col-0, *jmj15-3*, and *jmj15-4*) were sown on NaCl-infused media (1/2 MS medium with 0, 100, 150, and 200 mM NaCl, respectively). After stratification in darkness at 4°C for 2 days, the plates were transferred into a growth chamber under white light in 16 h light photoperiods. The germination (fully emerged radicle) rates were measured per day for a duration of 5 days. Each test was performed with more than three biological replicates.

### Root elongation assay

Measure root elongation under salt stress mainly according to the previous protocol (Lee and Zhu, 2009). More than 50 seedlings of Col-0 and *jmj15* mutation lines were sown on 1/2 MS medium. After stratification in darkness at 4°C for 2 days, the plates were moved to a vertical position in the growth chamber (16 h white light photoperiod, 20°C) for germination. The 4-day-old germinated seedlings were transferred onto vertical 1/2 MS agar plates supplemented with 0 m M NaCl, 50 m M NaCl, 100 mM NaCl and 150 mM NaCl, respectively. After 5 days with the salt treatment, the primary root length was measured and statistical significance was determined by two-sided *t*-test. Three replicates were performed for each line and treatment.

### RNA sequencing and data analysis

Ten-day-old wild type (Col-0) and *jmj15-4* mutants *Arabidopsis* plants treated with NaCl (0 mM or 150 mM NaCl solution in 1/2 MS liquid medium) for 5 h were pooled for RNA extraction and transcriptomic analysis. Plants harvested from three independent cultures were used as the biological replicates. Total RNA was extracted with Trizol (Invitrogen, Carlsbad, CA, United States), and treated with DNase I (Promega, Madison, WI, United States) to remove the genomic DNA. Then the mRNA was enriched by using oligo(dT) magnetic beads and broken into short fragments (200 bp) using the fragmentation buffer. The first strand cDNA was synthesized by using a random primer. RNase H, DNA polymerase I and dNTPs were used to synthesize the second strand. The double-strand cDNA was purified with magnetic beads. cDNA ends were repaired and a nucleotide A (adenine) was added at the 3′-end. Finally, PCR amplification was performed with fragments ligated by sequencing adaptors. The Agilent 2100 Bioanalyzer and the ABI Step One Plus real-time PCR system were used to qualify and quantify the QC library. The library products were sequenced with the Illumina HiSeqX-ten platform, and the library construction and sequencing were completed at Novogene Corporation (Tianjin, China). Gene expression was defined by RSEM (Li and Dewey, 2011) and estimated by FPKM. Differential expression analysis between two conditions/groups was performed using DESeq R package. The resulting *P*-values were adjusted using the Benjamini and Hochberg’s approach for controlling the false discovery rate (Anders and Huber, 2010). The RNA-seq data were deposited to NCBI-SRA databases under the accession PEJNA822702.

### Quantitative real-time RT-PCR

Total RNA was extracted from plant seedlings using Trizol (Invitrogen, Carlsbad, CA, United States) according to the manufacturer’s instructions. RNA concentration was measured by using a Nanodrop. For RT-qPCR analysis, 2 μg of total RNA treated with DNaseI (RQ1, Promega, M6101, Madison, WI, United States) were used to synthesize first-strand cDNA with Oligo(dT)15 primers using ImPromII reverse transcriptase (M3104A, Promega, M3104A, Madison, WI, United States). RT-qPCR was performed with LightCycler 480 SYBR Green I Master mix on the LightCycler 480 (Roche, Mannheim, Germany). The reactions were performed in triplicate for each run and at least three biological replicates were carried out for each reaction. Transcript levels were calculated using the comparative Ct (threshold cycle) method and utilizing *ACTIN2* as an internal control for data normalization. Primer sequences used in this study were summarized in Supplementary Table 1.

### Chromatin immunoprecipitation assay

Chromatin immunoprecipitation (ChIP) experiments were carried out as described previously with minor modifications (Shen et al., 2015). Ten-day-old plants were treated with NaCl (0 mM or 150 mM NaCl solution in 1/2 MS liquid medium) for 5 h before harvest. About 4 g of plant seedlings were harvested, which were then cross-linked with 1% formaldehyde for 10 min under vacuum and ground into fine powder in liquid nitrogen. The chromatin DNA was isolated and sonicated in the 200–1000 bp range with Diagenode Bioruptor UCD-300. The sonicated chromatin was pre-cleared and incubated with anti-H3K4me3 (Millipore, 07-473, Darmstadt, Germany), anti-RNAPII (Abcam, ab5408, Cambridge, United Kingdom) or anti-HA (Sigma, H6908, St. Louis, MO, United States) antibodies loaded Dynabeads™ protein A (Invitrogen, #10002D, Carlsbad, CA, United States) at 4°C overnight. Subsequently, the immunoprecipitated DNA was decrosslinked and purified by using the MinElute PCR Purification Kit (Qiagen, #28004, Hilden, Germany) according to the manufacturer’s instruction. ChIP DNA was used for sequencing or qPCR. ChIP-qPCR was performed with three biological replicates, and results were calculated as the percentage of input DNA. Sequences of the primers used for ChIP-qPCR were listed in Supplementary Table 1.

### ChIP-seq and data analysis

At least 5 ng of each ChIP DNA was used to construct ChIP-seq library, and two biological replicates for each sample. The Illumina libraries were constructed using the ChIP-seq DNA Sample Prep Kit (Illumina) according to manufacturer’s protocol. The ChIP-seq library was sequenced by Novogene Corporation (Tianjin, China) on the Illumina HiSeq2500 sequencing system. Base calling and read quality control were performed following the standard Illumina protocol. Reads passing quality control were aligned to the *Arabidopsis* genome (TAIR10^1^) using BWA (Burrows Wheeler Aligner) with default parameters and only uniquely mapped reads were kept (Li and Durbin, 2009). MACS2 (version 2.1.0) peak calling software was used to identify regions of enriched intervals over the background. A *q*-value threshold of 0.05 was used for all data sets. Differential enrichments were assessed by MACS2 (bdgdiff), with an FDR cutoff < 0.001. The differential histone modification regions were intersected with annotated genes to obtain the target genes using BED tools^2^. The alignments were converted to wiggle (WIG) files. The data were then imported into the Integrated Genome Browser for visualization. The ChIP-seq data were deposited to NCBI-SRA databases under the accession PRJNA823378.

## Results

### H3K4me3 dynamical change is associated with plant response to salt stress

**FIGURE 1:**
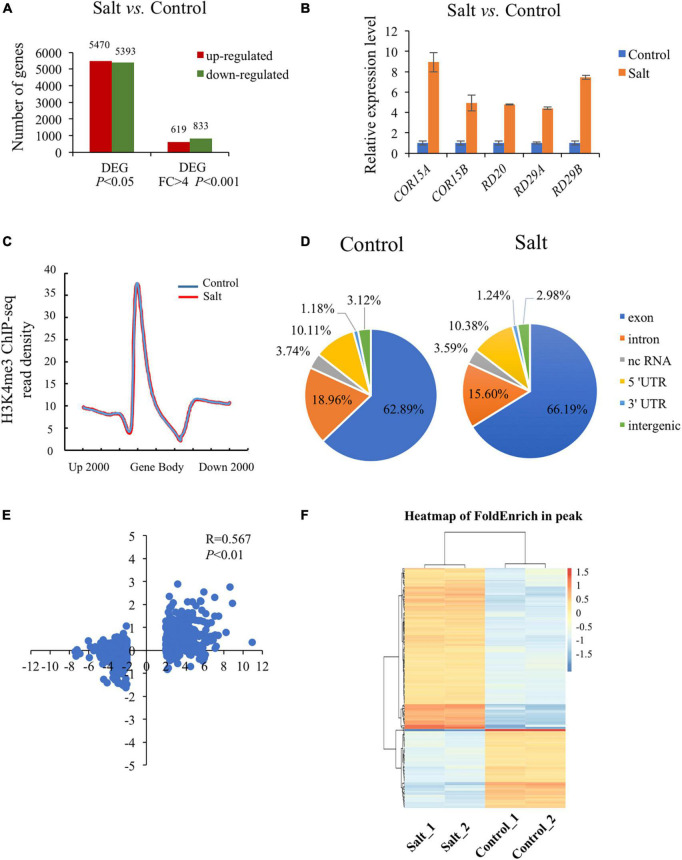
Global analysis of dynamic changes of H3K4me3 and mRNA levels during the plant response to salt stress. **(A)** Differently expressed genes in the wild type under salt condition compared with normal condition. The *Y*-axis represents the number of upregulated genes or downregulated genes by using the criteria *P* < 0.05 or fold change > 4, *P* < 0.001. **(B)** The mRNA level of 5 well known up-regulated genes which were strongly induced by salt stress. The data was calculated by log_2_(FPKM) and wild type under normal condition were set to 1. **(C)** Metaplot of the H3K4me3 distribution on genes in Col-0 under normal and salt stress conditions. Gene body in the *X*-axis represents all genes in the genome. Up 2000 represents 2000 bp upstream of Transcriptional Start Site (TSS) and down 2000 represents 2000 bp downstream of Transcriptional Terminal Site (TTS), respectively. The *Y*-axis represents read density of H3K4me3. **(D)** Genomic distribution of H3K4me3 peaks in Col-0 under normal and salt conditions. The definition of each region is described on the right. **(E)** Scatter plot of H3K4me3 and transcription changes of dysregulated genes in salt *vs.* normal (fold change > 4, *P* < 0.001). The horizontal axis represents the log_2_(fold change) of mRNA expression level. The vertical axis represents the log_2_(fold change) of H3K4me3 level. Pearson correlation indicates a correlation between the H3K4me3 and transcript dynamic changes under salt stress (*R* = 0.567, *P* < 0.01). **(F)** Heatmap shows the H3K4me3 enrichment of different peaks associated with genes under normal and salt condition (*P* < 0.05). The genes (rows) were subjected to hierarchical clustering. The color bar on the right indicates the Z-score.

To explore the transcriptional level of genes involved in plant response to salt stress, we conducted RNA-seq analysis using wild type seedlings under normal or salt-stressed conditions (0 mM or 150 mM NaCl solution in 1/2 MS liquid medium for 5 h), each with three biological replicates. The RNA-seq results showed that the three biological replicates were all highly correlated (*R* = 0.91–0.99). Analysis of differentially expressed genes (DEGs) revealed that 5470 up-regulated genes (URGs) and 5393 down-regulated genes (DRGs) in wild type seedlings as a result of the salt stress (*P* < 0.05) (Figure 1A and Supplementary Table 2). Using the strict criteria (fold change > 4.0, *P* < 0.001), 619 URGs and 833 DRGs were found in salt treated *versus* normal growth seedlings (Figure 1A and Supplementary Table 2). Among the genes up-regulated by salt stress, inducible genes implicated in salt stress response in other studies were present, including *COR15A* (AT2G42540), *COR15B* (AT2G42530), *RD20* (AT2G33380), *RD29A* (AT5G52310), *RD29B* (AT5G52300) (Figure 1B; Chen et al., 2010; Zheng et al., 2016; Yang et al., 2019).

**FIGURE 2:**
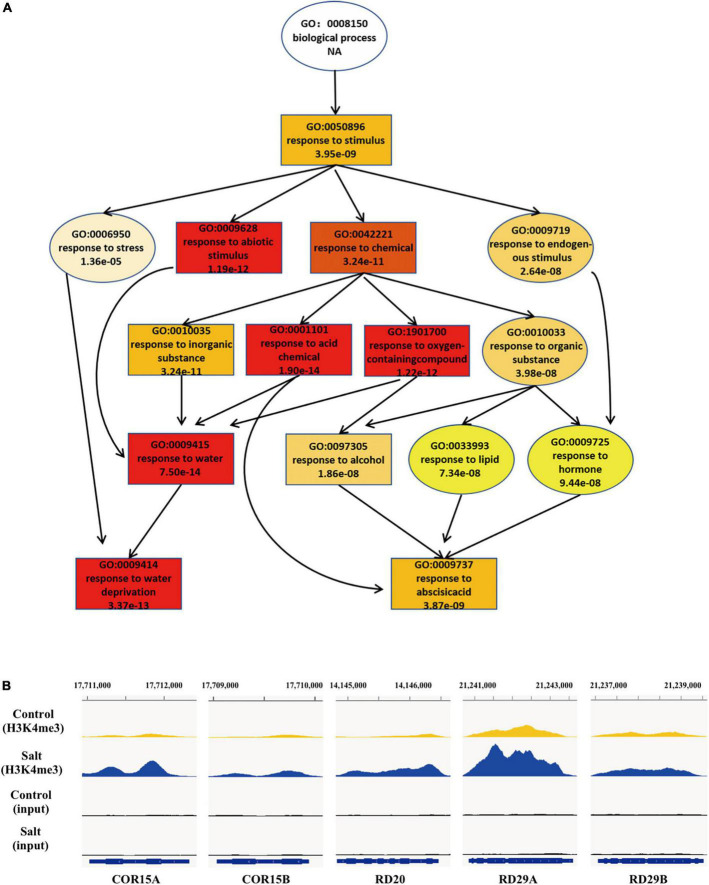
During the response to salt stress the H3K4me3 changes are associated with many of the genes involved in stress response. **(A)** A tree structure graph of the major significantly enriched GO terms of genes associated with different peaks (*P* < 0.05) of H3K4me3 between normal and salt conditions. GO terms are represented as boxes containing detailed description, including GO accession, description and *P*-value. **(B)** Integrative Genome Viewer (IGV) of H3K4me3 modifications at five representative salt-induced genes in Figure 1B under normal and salt-stressed conditions.

Increasing evidence has shown that transcriptional regulation directed by H3K4me3 is very important for environmental responses in plants (van Dijk et al., 2010; Huang et al., 2019; Foroozani et al., 2021). To reveal the possible regulatory role of H3K4me3 methylation in the plant response to salt stress, ChIP-seq was performed by using the wild type seedlings under normal and salt stress (the same conditions as the RNA-seq), each with two biological replicates. The results revealed that 21894 H3K4me3 methylation peaks in Col-0 samples under normal condition (Supplementary Table 3), while 24141 H3K4me3 peaks under salt stress treatment (Supplementary Table 4). The distribution of H3K4me3 was similar between normal and salt-treated seedlings, with the most enrichment occurring at the gene body (Figure 1C). In detail, most H3K4me3 amounts were found at exons (63–66%), followed by introns (16–19%) and 5′ untranslated regions (5′ UTR) (10%). Lower amounts of H3K4me3 were found at non-coding RNA (3.4%), intergenic region (3%) and 3′ UTR (1%) (Figure 1D), which was consistent with the previous data (Zhang et al., 2009; Huang et al., 2019). To determine whether/how salt stress-altered gene expression correlated with changes in H3K4me3, we examined the H3K4me3 changes for the 1452 genes with altered transcript abundance (|fold change| > 4.0, *P* < 0.001) (Supplementary Table 5). The plot analysis revealed a correlation between the H3K4me3 and transcript dynamic changes under salt stress (Pearson correlation coefficient *R* = 0.567, *P* < 0.01) (Figure 1E), indicating that H3K4me3 dynamics is associated with the transcript level of many genes involved in salt stress response. Totals of 2591 and 1905 genes were differentially hypermethylated and hypomethylated between salt and normal conditions (*P* < 0.05), respectively (Figure 1F and Supplementary Table 6). Gene ontology (GO) enrichment analysis revealed that the H3K4me3 changed genes under salt stress were categorized into ‘response to water deprivation,’ ‘response to abiotic stimulus,’ ‘response to abscisic acid,’ and ‘response to chemical’ (Figure 2A). In addition, the up-regulated salt-responsive genes (i.e., *COR15A*, *COR15B*, *RD20*, *RD29A*, *RD29B*) were found hypermethylated under salt stress condition by using the Integrated Genome Browser (Figure 2B). These results suggest that the H3K4me3 change is associated with the transcriptional change of the salt responsive genes during the stress process.

### *jmj15* loss-of-function display hypersensitivity to salinity stress

**FIGURE 3:**
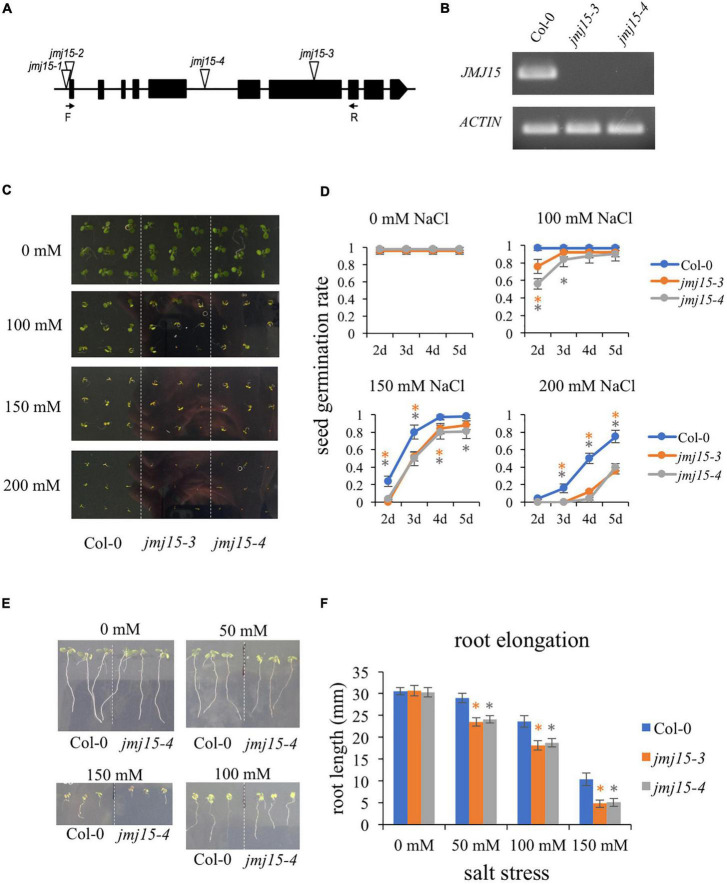
Loss-of-function of *JMJ15* in *Arabidopsis thaliana* are hypersensitive to salt stress. **(A)** Schematic diagram of the *JMJ15* gene. Exons are represented by black boxes. Triangles indicate T-DNA insertions. The primer positions of forward (F) and reverse (R) are indicated by arrows. **(B)** Reverse transcription polymerase chain reaction (RT-PCR) analysis of transcripts of Col-0, *jmj15-3* and *jmj15-4* by using the indicated primers. *ACTIN2* was used as an internal control. **(C,D)** Measurement of seed germination rates. **(C)** Images of the wild type, *jmj15-3* and *jmj15-4* seeds germinated on 1/2 MS medium supplemented with or without indicated concentrations of NaCl for 5 days. **(D)** Quantification of seed germination rates of genotypes shown in **(C)** at different time points. Data represent means of ± SD (*n* = 3, three biological replicates were performed with at least 100 seeds per genotype in each replicate). **(E,F)** Measurement of root elongation. **(E)** Wild-type and *jmj15* seedlings were grown on vertical 1/2 MS plate for 4 days and then transfered onto vertical 1/2 MS medium supplemented with or without different concentration of NaCl for 5 days. **(F)** The primary root length was measured after 5 days with different indicated concentrations of NaCl. Data represent means of ± SD (*n* = 3, three biological replicates were performed with at least 50 seedlings per genotype in each replicate). Asterisks indicate where the difference between Col-0 and mutant is statistically significant (Student’s *t*-test, *P < 0.05).

We have shown that gain-of-function mutants (*jmj15-1* and *jmj15-2*) exhibited increased tolerance to salinity stress, whereas one allele of loss-of-function mutant (*jmj15-3*) exhibited increased sensitivity to salinity stress (Shen et al., 2014). To further confirm the response of *jmj15* loss-of-function mutant to salt stress, we obtained another mutant allele *jmj15-4*. In *jmj15-4*, T-DNA was inserted in the 5th intron of *JMJ15* (Figure 3A). RT-qPCR analysis revealed that the *JMJ15* transcripts were abolished in both *jmj15-3* and *jmj15-4* alleles (Figure 3B). Both loss-of-function lines together with the wild type (Col-0) were tested for germination rates on normal MS media and MS supplemented with different salt concentrations. The germination rates were scored at different time points after 2 days. Seeds from three various harvests were tested. Under normal conditions, the germination rates of wild type and *jmj15* loss-of-function mutants were more than 95%. When treated with salt, the germination rate of *jmj15* loss-of-function mutants were lower than the wild type at different indicated salt concentration and time points (Figures 3C,D). Root elongation lengths were also measured to analyze the sensitivity of plants to the salt stress. The results revealed that after 5 days under normal condition, the root lengths of loss-of-function *jmj15* and wild type plants were similar. However, the root lengths of *jmj15* mutants were shorter than that of wild type after 5 days under various concentration of NaCl treatment (Figures 3E,F). Together, these results suggest that JMJ15 regulates the response to salt stress in *Arabidopsis*.

### The loss-of-function *jmj15* mutants impaired the salt responsive gene expression program

**FIGURE 4:**
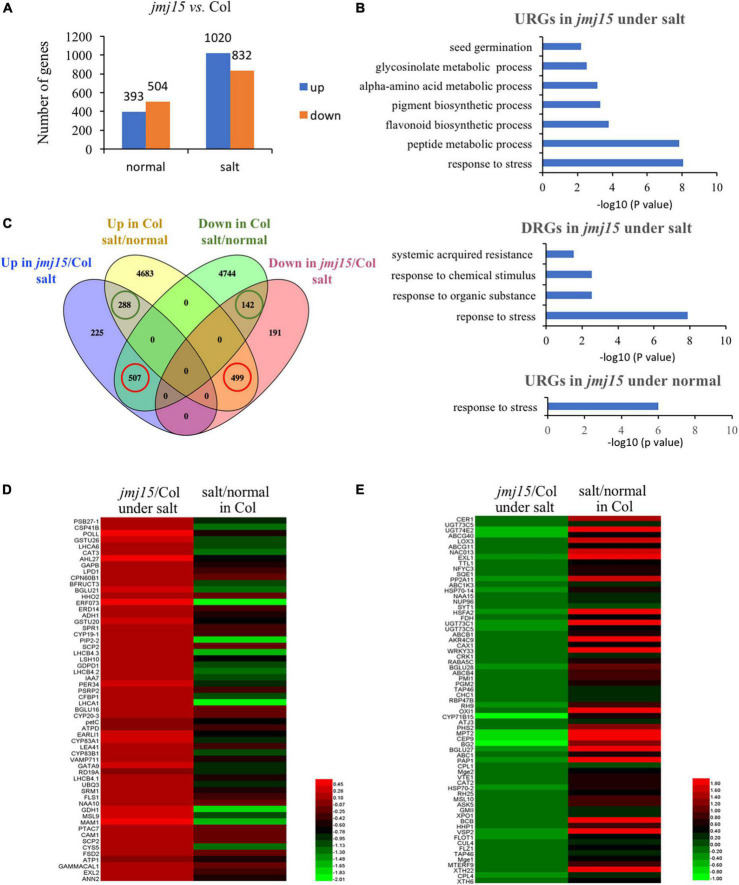
Transcriptomic analysis of *jmj15* loss-of-function mutants compared to Col-0 under normal and salt-stressed conditions. **(A)** Differently expressed genes in the wild type and *jmj15* mutations under normal and salt conditions. The *Y*-axis represents the number of upregulated genes or downregulated genes by using the criteria *P* < 0.05. **(B)** GO term enrichment of the upregulated genes (URGs) and downregulated genes (DRGs) in *jmj15* mutants at the salt and normal conditions. **(C)** Venn diagram of comparison of DEGs in *jmj15-4* (relative to Col-0 under salt condition) and DEGs in Col-0 (under salt relative to normal condition). The green cycles represent the number of genes that were up-regulated or down-regulated both in *jmj15*/Col-0 under salt condition and Col-0 under salt/normal. The red cycles represent the number of genes that were up-regulated in *jmj15*/Col-0 under salt condition but down-regulated in Col-0 under salt/normal, or down-regulated in *jmj15*/Col-0 under salt condition but up-regulated in Col-0 under salt/normal. **(D,E)** Heatmaps show the expression of the misregulated stress-related genes (GO: 0006950) in *jmj15*/Col-0 and salt/normal (red cycles in **C**). **(D)** Shows URGs in *jmj15*/Col-0 and DRGs under salt/normal; **(E)** shows DRGs in *jmj15*/Col-0 and URGs under salt/normal.

The developmental phenotype between *jmj15-4* and wild type was highly significant different under salt stress (Figure 3). To explore the genome-wide transcriptional landscape directed by JMJ15, we conducted RNA-seq analysis of *jmj15* loss-of-mutation (*jmj15-4*) and Col-0 under normal and salt stress conditions, each with three replicates. The total of 897 and 1852 DEGs were identified in *jmj15-4* compared with Col-0 under normal and salt stress conditions, respectively (*P* < 0.05) (Figure 4A). Among 897 DEGs in *jmj15-4* under normal condition, 393 were upregulated (URGs) and 504 were downregulated (DRGs) in *jmj15-4* (Figure 4A and Supplementary Table 7), while among 1852 DEGs under salt stress, 1020 were URGs and 832 were DRGs in *jmj15-4* (Figure 4A and Supplementary Table 8), suggesting that JMJ15 may mainly play a role as a transcriptional repressor during the salt responsive process. GO enrichment revealed that the URGs in *jmj15*/Col-0 under salt stress were enriched in response to stress, peptide metabolic process, flavonoid metabolic process, pigment synthetic process, and the DRGs were enriched in response to stress, response to organic substance, response to chemical stimulus, systematic acquired resistance (Figure 4B). URGs in *jmj15*/Col-0 under normal condition were enriched in response to stress, but no GO enrichment for DRGs (Figure 4B). These results suggest that loss of *JMJ15* may mostly affect metabolism and the responsiveness to environmental stimuli of seedlings which is consistent with the previous result that overexpression of *JMJ15* preferentially represses the stress regulatory genes (Shen et al., 2014).

To investigate how the *jmj15* mutation affects the salt responsive gene expression program, we compared DEGs in *jmj15* mutation versus Col-0 under salt treatment (*jmj15*/Col-0 under salt) with that of Col-0 under salt versus normal condition (salt/normal in Col-0). Interestingly, we found that (507/1020, 49.7%) upregulated and (499/832, 55.6%) downregulated DEGs in *jmj15*/Col-0 under salt stress overlapped with the downregulated and upregulated DEGs in Col-0 under salt/normal respectively (Figure 4C red cycle and Supplementary Table 9). However, the overlap was much less between the upregulated (288/1020, 28.2%) and downregulated (142/832, 17.1%) DEGs in *jmj15*/Col-0 under salt stress with the upregulated and downregulated DEGs in Col-0 under salt/normal (Figure 4C green cycle). These data indicated that *jmj15* mutation impaired the salt responsive gene expression program, that may explain the salt-stress-sensitive phenotype in *jmj15* loss-of-function mutation. Further investigation of these 507 genes that were upregulated in *jmj15*/Col-0 under salt but downregulated in Col-0 under salt/normal found some key components of plant tolerance to stress, such as *PRXIIF* (AT3G06050), *ERF73* (AT1G72360), *PIP2B* (AT2G37170), *CAM1* (AT5G37780), *LTP5* (AT3G51600), *PDF1.2* (AT5G44420), *PDF1.2B* (AT2G26020), *PDF1.3* (AT2G26010), *LEA41*/*DI21* (AT4G15910), *SAP18* (AT2G45640), *ERD14* (AT1G76180), *SESA* (AT4G27170), *EARLI1* (AT4G12480), *GSTF9* (AT2G30860), *GSTU26* (AT1G17190), *GSTU20* (AT1G78370) (Figure 4D and Supplementary Table 9). The result suggested that loss of *JMJ15* derepressed the expression of some stress-responsive genes, which was inhibited during the salt treatment. Similarly, we found some stress related genes such as *CAT2* (AT1G58030), *XTH6* (AT5G65730), *XTH22*/*TCH4* (AT5G57560), *HSFA2* (AT2G26150), *NRT2.6* (AT3G45060), *HSP70-2* (AT5G02490), *COR15A* (AT2G42540), *COR15B* (AT2G42530), *NAC13* (AT1G32870), *VSP2* (AT5G24770) that were upregulated in Col-0 under salt/normal but downregulated in *jmj15*/Col-0 under salt stress (Figure 4E and Supplementary Table 9). Together, the analysis suggested that JMJ15 was required for both gene activation and repression of the salt-responsive gene expression program.

### JMJ15 regulates H3K4me3 levels of many salt responsive genes

**FIGURE 5:**
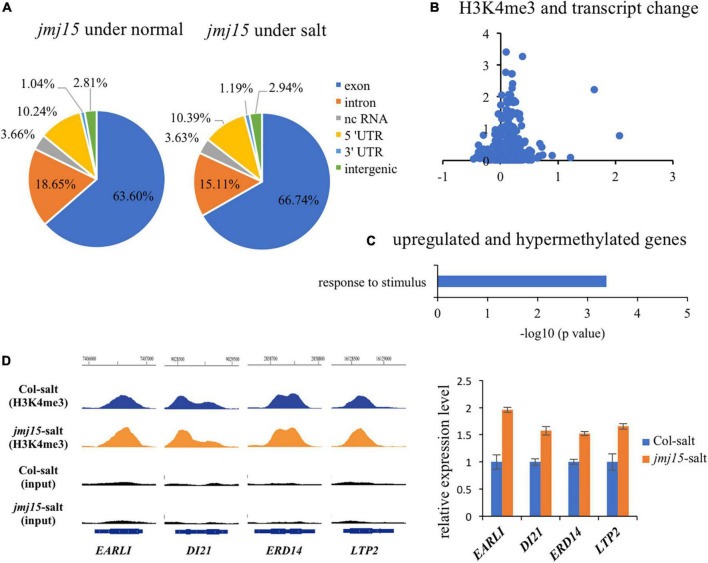
JMJ15 affects H3K4me3 levels at many stress-related genes under salt stress. **(A)** Genomic distribution of H3K4me3 peaks in *jmj15-4* under normal and salt conditions. **(B)** Scatterplot shows that most of up-regulated genes of *jmj15* mutation under salt stress were H3K4me3 hypermethylated. The *x*-axis represents the log_2_(fold change) of H3K4me3 level in *jmj15* compared to Col-0 under salt stress. The *y*-axis represents the log_2_(fold change) of mRNA level in *jmj15* compared to Col-0 under salt stress. **(C)** GO term enrichment of the genes upregulated and H3K4me3 hypermethylated in *jmj15* mutants compared to Col-0 under salt stress. **(D)** H3K4me3 and mRNA level of selective stress-related genes in *jmj15* mutants under salt stress. Left, IGV showing H3K4me3 levels of stress-related genes in *jmj15* mutants under salt stress. Right, the transcript level in *jmj15* mutants under salt stress. The data was calculated by log_2_(FPKM) and wild type were set to 1.

To investigate whether JMJ15 is involved in the salt stress response by demethylating H3K4me3, we examined H3K4me3 patterns in the *jmj15* loss-of-function mutants under normal and salt-stressed conditions by ChIP-seq. The result revealed that 21964 H3K4me3 methylation peaks in *jmj15* mutation under normal condition (Supplementary Table 10), while 24273 H3K4me3 methylation peaks under salt stress treatment (Supplementary Table 11). The distribution pattern is similar in *jmj15* to WT under both conditions (Figures 1D, 5A). However, totals of 722 and 1763 genes were differentially hypermethylated and hypomethylated in the *jmj15* loss-of-function mutants under salt stress, respectively (*P* < 0.05) (Supplementary Table 12). Scatterplot analysis of increased expressed genes in *jmj15*/Col-0 under salt showed that most transcriptionally URGs were hypermethylated (Figure 5B and Supplementary Table 13). GO enrichment analysis of these hypermethylated and URGs in *jmj15* under salt was the most enriched in response to stimulus (Figure 5C). By using Integrated Genome Browser, the H3K4me3 level was increased for some stimulus responsive genes such as *EARLI* (AT4G12480), *DI21* (AT4G15910), *ERD14* (AT1G76180) and *LTP2* (AT2G38530) (Figure 5D and Supplementary Table 13), suggesting that the increased transcription of these stress related genes may be due to decreased demethylation activity of H3K4me3 in the *jmj15* mutants. Of note, we found some transcriptional factors that both the transcription and H3K4me3 levels increased in *jmj15* knockout mutations under salt condition such as some stress-responsive *WRKY* genes and ethylene-responsive-element binding factor genes (Supplementary Table 14), consisting with our previous data that overexpression of *JMJ15* preferentially represses the stress regulatory genes, especially some transcription factors.

### JMJ15 controls the H3K4me3 levels of *WRKY46* and *WRKY70* in response to salt stress

**FIGURE 6:**
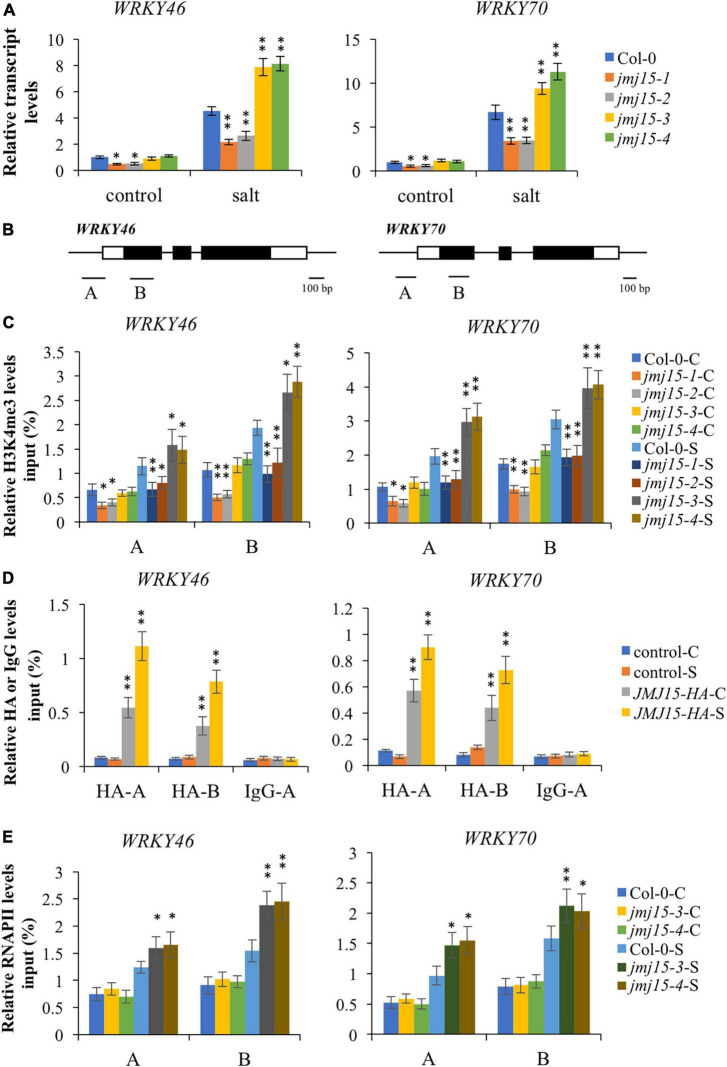
Transcript and chromatin changes at the *WRKY46* and *WRKY70* loci in *jmj15* mutations. **(A)** RT-qPCR analysis of the transcript level of *WRKY46* and *WRKY70* genes in *jmj15* mutants compared to Col-0 under normal and salt conditions. *ACTIN2* was used as an internal control to normalize the gene expression level. The wild type under normal condition were set to 1. Bars are means ± SD of data from three biological replicates. *P < 0.05 and **P < 0.001, Student’s *t*-test. **(B)** Schematic diagram of *WRKY46* and *WRKY70*. Regions A and B of *WRKY* genes were used for ChIP-qPCR analysis. Black bar represents 100 bp. **(C)** ChIP-qPCR analysis of H3K4me3 of *WRKY46* and *WRKY70* genes in Col-0 and *jmj15* mutants under control condition (C) and salt condition (S). Results are presented as percentage of input. Bars are means ± SD of data from three biological replicates. *P < 0.05 and **P < 0.001, Student’s *t*-test. **(D)** JMJ15-HA ChIP-qPCR was performed by using HA antibody to determine the occupancy of JMJ15-HA at the *WRKY46* and *WRKY70* loci under control condition (C) and salt condition (S). IgG was used as a negative control. Results are presented as percentage of input. Bars are means ± SD from three biological replicates. *P < 0.05 and **P < 0.001, Student’s *t*-test. **(E)** ChIP-qPCR analysis of occupancy of RNA polymerase II (RNAPII) at the *WRKY46* and *WRKY70* loci in Col-0 and *jmj15* loss-of-function mutants under control condition (C) and salt stress condition (S). Results are presented as percentage of input. Bars are means ± SD from three biological replicates. *P < 0.05 and **P < 0.001, Student’s *t*-test.

Interestingly, among these hypermethylated and URGs in *jmj15* under salt stress, we found two known transcription factors, *WRKY46* and *WRKY70*, that function as negative regulators in abiotic stress signaling in plants (Li et al., 2013; Ding et al., 2015; Chen et al., 2017). WRKY46 modulates the lateral root development in salt stress condition via regulation of ABA signaling and auxin homeostasis (Ding et al., 2015). WRKY70⋅modulates osmotic stress response by regulating stomatal aperture in *Arabidopsis* (Li et al., 2013). Meanwhile, WRKY70 was shown to be a direct target of ATX1 and positively regulated by ATX1-generated H3K4me3 (Alvarez-Venegas et al., 2007). Therefore, we hypothesized that JMJ15 might be involved in salt stress responses by modulating the H3K4me3 level of *WRKY46* and *WRKY70* genes. First, we examined the transcript level of these two *WRKY* genes in the *jmj15* loss-of-function mutants (*jmj15-3* and *jmj15-4*), *jmj15* gain-of-function mutants (*jmj15-1* and *jmj15-2*) and wild type (Col-0) under normal and salt-stressed conditions. The result showed that the transcript level of *WRKY46* and *WRKY70* was induced in wild type by salt stress. In *jmj15* loss-of-function mutants, the transcript level of these two *WRKY* genes was enhanced compared to wild type under salt treatment, whereas the transcript level was obviously decreased in *jmj15* gain-of-function mutations compared to wild type under salt treatment (Figure 6A). However, no discernible alterations were detected in *jmj15* loss-of-function mutants under normal condition, but the transcript level of two *WRKY* genes were declined in *jmj15* gain-of-function mutants under normal condition (Figure 6A), which was consistent with our previous result that overexpression of JMJ15 inhibited the expression of some transcription factors including some *WRKY* genes under normal condition (Shen et al., 2014). To confirm the role of JMJ15 in regulating chromatin status at the loci of *WRKY46* and *WRKY70* during the salt responsive process, we performed ChIP-qPCR analysis of the *jmj15* loss-of-function mutants and *jmj15* gain-of-function mutants (Figure 6B). The results showed that H3K4me3 level of *WRKY46* and *WRKY70* were obviously increased in *jmj15-3* and *jmj15-4* compared with wild type under salt stress, but decreased in *jmj15-1* and *jmj15-2* compared to wild type under normal and salt stress conditions (Figure 6C), suggesting that JMJ15 regulates the transcript levels of *WRKY46* and *WRKY70* genes by modulating their H3K4me3 levels.

To investigate whether JMJ15 is directly associated with *WRKY46* and *WRKY70* genes, we analyzed the *35S-JMJ15-HA* plants by ChIP-qPCR using anti-HA antibody (Shen et al., 2014). The analysis revealed that JMJ15-HA was enriched in *WRKY46* and *WRKY70* relative to the negative control (IgG), and JMJ15-HA binding levels were higher at the promoter and gene body under the salt stress compared to normal condition (Figure 6D). The results suggest that JMJ15 is recruited to the *WRKY46* and *WRKY70* genes and regulates their expression during the salt stress process. It has been established that H3K4me3 levels have a positive correlation with transcription rates and occupancy of activated RNA polymerase II (RNAPII) (Ardehali et al., 2011). We then tested whether the RNAPII occupancy at *WRKY46* and *WRKY70* was affected by JMJ15 following salt treatment. The results revealed that binding of RNAPII to *WRKY46* and *WRKY70* were increased in *jmj15* loss-of-function mutants compared to wild type under salt stress (Figure 6E). Taken together, these results indicate that JMJ15 modulates H3K4me3 levels at the *WRKY46* and *WRKY70* loci and thus in turn affects RNAPII occupancy, which is essential for gene expression.

## Discussion

### H3K4me3 dynamics impact the abiotic stress response in plants

H3K4me3 has been implicated in the regulation of a number of environmental responses, such as drought stress, heat stress, submergence stress, and salt stress. For example, H3K4me3 abundance along with transcript levels of the inducible drought marker genes (*RD29A*, *RD29B*, *RD20*, and *RAP2.4*) were increased in response to drought (Kim et al., 2008). The genome-wide studies also showed that many genes up- or down-regulated by dehydration exhibited the similar increased or decreased H3K4me3 abundance, respectively (van Dijk et al., 2010; Zong et al., 2013). ATX1, H3K4me3 methyltransferase, was reported to involve in drought stress by affecting biosynthesis of ABA resulting from regulating H3K4me3 and transcript of *NCED3* (Ding et al., 2011). Investigation of a number of stress-responsive genes in *Arabidopsis* has shown that several chromatin marks impact the responsiveness of salt-induced genes. For instance, salt stress led to enrichment of H3K4me3 and H3K9K14 acetylation, but decreased H3K9me2 at a subset of salt stress-responsive loci (Chen et al., 2010). Histone deacetylase 6 (HDA6) and HDA9 led to increased H3 acetylation at salt-inducible genes in *hda6* mutants compared to wild type (Chen et al., 2010; Zheng et al., 2016). Interestingly, mutation of HDA6 also abolished H3K4me3 enrichment of ABA-induced loci (Chen et al., 2010). A recent study showed that salt stress altered the distribution of H3K4me3 in a tissue specific manner. In response to salt, rice seedlings exhibited H3K4me3 decreases and increases in exons and introns respectively, whereas only exons decrease of H3K4me3 in roots (Zheng et al., 2019). In our study, the distribution pattern of H3K4me3 was similar between normal and salt-treated seedlings. However, when compared the H3K4me3 level and transcript dynamic abundance for some transcript changed genes (| fold change| > 4.0), there was a correlation between the H3K4me3 and transcript dynamic changes under salt stress (Figure 1E). In addition, the H3K4me3 peak difference associated genes under salt stress were enriched in response to stress (Figure 2). These results indicate that H3K4me3 dynamics is associated with the transcript level of many genes involved in salt stress response.

### KDM5/JARID group proteins differently response to abiotic stress

In humans, KDM5/JARID group proteins are active histone H3K4me1/2/3 demethylases (Lu et al., 2008; Sun and Zhou, 2008). In *Arabidopsis*, six proteins (JMJ14-19) have been identified as members of this group based on phylogenetic analysis. Among these, JMJ14, JMJ15, JMJ16, JMJ18 harbor JmjN, JmjC, C5HC2, FY-rich N-terminus (FYRN), and FY-rich C-terminus (FYRC) domains. By contrast, JMJ19 harbors only JmjN, JmjC, and C5HC2 domains, whereas JMJ17 harbors JmjC, zf-C5HC2, PLU-1, and PHD domains (Lu et al., 2008). JMJ14, JMJ15, and JMJ18 play important roles in regulating flowering by reducing H3K4me3 abundance at various flowering regulatory genes. In addition to regulating the transition to flowering, *Arabidopsis* demethylases also play roles in RNA silencing (Searle et al., 2010); circadian clock (Song et al., 2019); defense (Li et al., 2020); senescence (Liu et al., 2019); de-etiolation process (Islam et al., 2021), and dehydration stress (Huang et al., 2019). JMJ17 played a role in dehydration stress by directly binding to *OST1* and demethylating H3K4me3 levels (Huang et al., 2019). JMJ17 also regulates ABA responsive genes by interacting with WRKY40 (Wang et al., 2021). Our data showed that JMJ15 involved in salt stress by directly binding to the chromatin and regulating the expression of *WRKY46* and *WRKY70* genes. It seems that JMJ17 did not function in salt stress response as *jmj17/jmj15* double mutations showed the similar salt stress response phenotype as *jmj15* single mutant while *jmj17* single mutant did not show discernible phenotype of salt stress response compared to wild type (Huang et al., 2019). These results indicate that these two KDM5/JARID histone demethylases function in different physiological processes without functional redundancy, which might be explained by their existing different domains and various target genes.

### JMJ15 functions in salt stress tolerance by regulating *WRKY* genes

WRKY transcription factors, as a large family of plant transcription factors, participate in a variety of biological processes, including root growth, the quality of blossom clusters, senescence of leaf, fruit maturation, resistance to pathogens, abiotic stress response (Chen et al., 2017; Viana et al., 2018; Wang et al., 2020; de Bont et al., 2022; Goyal et al., 2022). *WRKY* gene family members were categorized into three groups (Rushton et al., 2010). WRKY46 and WRKY70 belonged to the group III with one WRKY domain and CCHC zinc finger motif, which played critical roles in the regulation of biotic stress response (Li et al., 2006; Hu et al., 2012). Furthermore, WRKY46 and WRKY70 acted as essential regulators in abiotic stress response which had also been demonstrated (Li et al., 2013; Ding et al., 2014; Chen et al., 2017). WRKY46 was found to negatively regulate plant responses to abiotic stress as the overexpression of *WRKY46* resulted in hypersensitivity to drought and salt stress with a higher rate of water loss (Ding et al., 2014). Similarly, WRKY70 functions as negative regulator in drought stress and osmotic stress response by affecting water retention and stomatal conductance (Li et al., 2013; Chen et al., 2017). In our study, we found that the expression of *WRKY46* and *WRKY70* were negatively regulated by JMJ15 under salt stress (Figure 6A), which was consistent with the hypersensitivity of *JMJ15* loss-of-function mutants to salt stress response. Furthermore, we found that JMJ15 bound to the chromatin of *WRKY46* and *WRKY70*, and the enrichment was slightly induced under salt stress treatment (Figure 6D). Notably, overexpression of *JMJ15* led to decrease the transcript levels of *WRKY* genes as well as H3K4me3 levels at these loci. According to publicly available microarray data^3^ (Winter et al., 2007), the expression of *JMJ15* was low but rapidly induced by salt treatment (0.5–1 h), while *WRKY46* and *WRKY70* were induced at later time (3–6 h) (Supplementary Figure 1). These data suggest that JMJ15 may mainly repress the gene expression of *WRKY46* and *WRKY70* at the early stage of salt stress. Our results showed that H3K4me3 levels increased substantially at the *WRKY46* and *WRKY70* genes after 5 h salt treatment. This result suggests the existence of H3K4me3 methyltransferases and other chromatin remodelers that may also modulate the chromatin of *WRKY46* and *WRKY70* genes during the salt stress process. WRKY70 was previously reported as one target gene of H3K4me3 histone methyltransferase ATX1 (Ding et al., 2011, 2012). The SUMO E3 ligase, SIZ1 was found to modulate transcript and H3K4me3 level at the *WRKY70* gene (Miura et al., 2020). However, it is not excluded other chromatin remodelers regulate H3K4me3 abundance and the transcript levels of *WRKY46* and *WRKY70* genes. Further studies should be conducted to understand how JMJ15 cooperates with other chromatin remodelers to regulate target genes.

## Data availability statement

The data presented in the study are deposited in the NCBI-SRA repository, accession number PRJNA822702 https://www.ncbi.nlm.nih.gov/bioproject/PRJNA822702 and PRJNA823378 https://www.ncbi.nlm.nih.gov/bioproject/PRJNA823378.

## Author contributions

YS, YC, and LS designed the research and analyzed the data. YS, YC, SL, HL, and LS performed the experiments. YS and LS wrote the manuscript. All authors have read and approved the final manuscript.

## Footnotes

1
www.arabidopsis.org

2
https://bedtools.readthedocs.io/en/latest/content/installation.html

3
http://bbc.botany.utoronto.ca/efp

## Supplementary material

Supplementary Figure 1 Expression patterns of *JMJ15*, *WRKY46*, and *WRKY70* under 150 mM NaCl treatment for 24 h. The expression data were obtained from a public database, eFP browser (http://bar.utoronto.ca/efp_arabidopsis/cgi-bin/efpWeb.cgi). The color bar on the right indicates the absolute expression values.

## References

[B1] Alvarez-Venegas R. Abdallat A. A. Guo M. Alfano J. R. Avramova Z. (2007). Epigenetic control of a transcription factor at the cross section of two antagonistic pathways. *Epigenetics* 2 106–113. 10.4161/epi.2.2.4404 17965588

[B2] Anders S. Huber W. (2010). Differential expression analysis for sequence count data. *Genome. Biol.* 11:R106. 10.1186/gb-2010-11-10-r106 20979621 PMC3218662

[B3] Ardehali M. B. Mei A. Zobeck K. L. Caron M. Lis J. T. Kusch T. (2011). Drosophila Set1 is the major histone H3 lysine 4 trimethyltransferase with role in transcription. *EMBO J.* 30 2817–2828. 10.1038/emboj.2011.194 21694722 PMC3160253

[B4] Chen J. Nolan T. M. Ye H. Zhang M. Tong H. Xin P. (2017). Arabidopsis WRKY46, WRKY54, and WRKY70 transcription factors are involved in brassinosteroid-regulated plant growth and drought responses. *Plant Cell* 29 1425–1439. 10.1105/tpc.17.00364 28576847 PMC5502465

[B5] Chen L. T. Luo M. Wang Y. Y. Wu K. (2010). Involvement of arabidopsis histone deacetylase HDA6 in ABA and salt stress response. *J. Exp. Bot.* 61 3345–3353. 10.1093/jxb/erq154 20519338 PMC2905197

[B6] Chen X. Hu Y. Zhou D. X. (2011). Epigenetic gene regulation by plant Jumonji group of histone demethylase. *Biochim. Biophys. Acta* 1809 421–426. 10.1016/j.bbagrm.2011.03.004 21419882

[B7] de Bont L. Mu X. Wei B. Han Y. (2022). Abiotic stress-triggered oxidative challenges: Where does H2S act? *J. Genet. Genom*. 49 748–755. 10.1016/j.jgg.2022.02.019 35276389

[B8] Ding Y. Avramova Z. Fromm M. (2011). The Arabidopsis trithorax-like factor ATX1 functions in dehydration stress responses via ABA-dependent and ABA-independent pathways. *Plant J.* 66 735–744. 10.1111/j.1365-313X.2011.04534.x 21309869

[B9] Ding Y. Fromm M. Avramova Z. (2012). Multiple exposures to drought ‘train’ transcriptional responses in Arabidopsis. *Nat. Commun.* 3:740. 10.1038/ncomms1732 22415831

[B10] Ding Z. J. Yan J. Y. Li C. X. Li G. X. Wu Y. R. Zheng S. J. (2015). Transcription factor WRKY46 modulates the development of Arabidopsis lateral roots in osmotic/salt stress conditions via regulation of ABA signaling and auxin homeostasis. *Plant J.* 84 56–69. 10.1111/tpj.12958 26252246

[B11] Ding Z. J. Yan J. Y. Xu X. Y. Yu D. Q. Li G. X. Zhang S. Q. (2014). Transcription factor WRKY46 regulates osmotic stress responses and stomatal movement independently in Arabidopsis. *Plant J.* 79 13–27. 10.1111/tpj.12538 24773321

[B12] Foroozani M. Vandal M. P. Smith A. P. (2021). H3K4 trimethylation dynamics impact diverse developmental and environmental responses in plants. *Planta* 253:4. 10.1007/s00425-020-03520-0 33387051

[B13] Goyal P. Devi R. Verma B. Hussain S. Arora P. Tabassum R. (2022). WRKY transcription factors: Evolution, regulation, and functional diversity in plants. *Protoplasma* [Epub ahead of print]. 10.1007/s00709-022-01794-7 35829836

[B14] Hu Y. Dong Q. Yu D. (2012). Arabidopsis WRKY46 coordinates with WRKY70 and WRKY53 in basal resistance against pathogen *Pseudomonas syringae*. *Plant Sci.* 185-186 288–297. 10.1016/j.plantsci.2011.12.003 22325892

[B15] Hu Y. Shen Y. Conde E. S. N. Zhou D. X. (2011). The role of histone methylation and H2A.Z occupancy during rapid activation of ethylene responsive genes. *PLoS One* 6:e28224. 10.1371/journal.pone.0028224 22140554 PMC3225391

[B16] Huang S. Zhang A. Jin J. B. Zhao B. Wang T. J. Wu Y. (2019). Arabidopsis histone H3K4 demethylase JMJ17 functions in dehydration stress response. *New Phytol.* 223 1372–1387. 10.1111/nph.15874 31038749

[B17] Islam M. T. Wang L. C. Chen I. J. Lo K. L. Lo W. S. (2021). Arabidopsis JMJ17 promotes cotyledon greening during de-etiolation by repressing genes involved in tetrapyrrole biosynthesis in etiolated seedlings. *New Phytol.* 231 1023–1039. 10.1111/nph.17327 33666236

[B18] Jaskiewicz M. Conrath U. Peterhansel C. (2011). Chromatin modification acts as a memory for systemic acquired resistance in the plant stress response. *EMBO Rep.* 12 50–55. 10.1038/embor.2010.186 21132017 PMC3024125

[B19] Jeong J. H. Song H. R. Ko J. H. Jeong Y. M. Kwon Y. E. Seol J. H. (2009). Repression of flowering locus T chromatin by functionally redundant histone H3 lysine 4 demethylases in Arabidopsis. *PLoS One* 4:e8033. 10.1371/journal.pone.0008033 19946624 PMC2777508

[B20] Kim J. M. Sasaki T. Ueda M. Sako K. Seki M. (2015). Chromatin changes in response to drought, salinity, heat, and cold stresses in plants. *Front. Plant Sci.* 6:114. 10.3389/fpls.2015.00114 25784920 PMC4345800

[B21] Kim J. M. To T. K. Ishida J. Matsui A. Kimura H. Seki M. (2012). Transition of chromatin status during the process of recovery from drought stress in *Arabidopsis thaliana*. *Plant Cell Physiol.* 53 847–856. 10.1093/pcp/pcs053 22505693

[B22] Kim J. M. To T. K. Ishida J. Morosawa T. Kawashima M. Matsui A. (2008). Alterations of lysine modifications on the histone H3 N-tail under drought stress conditions in *Arabidopsis thaliana*. *Plant Cell Physiol.* 49 1580–1588. 10.1093/pcp/pcn133 18779215

[B23] Lee B. H. Zhu J. K. (2009). Phenotypic analysis of *Arabidopsis* mutants: Root elongation under salt/hormone-induced stress. *Cold Spring Harb. Protoc.* 2009:dbrot4968. 10.1101/pdb.prot4968 20150051

[B24] Li B. Carey M. Workman J. L. (2007). The role of chromatin during transcription. *Cell* 128 707–719. 10.1016/j.cell.2007.01.015 17320508

[B25] Li B. Dewey C. N. (2011). RSEM: Accurate transcript quantification from RNA-Seq data with or without a reference genome. *BMC Bioinform.* 12:323. 10.1186/1471-2105-12-323 21816040 PMC3163565

[B26] Li D. Liu R. Singh D. Yuan X. Kachroo P. Raina R. (2020). JMJ14 encoded H3K4 demethylase modulates immune responses by regulating defence gene expression and pipecolic acid levels. *New Phytol.* 225 2108–2121. 10.1111/nph.16270 31622519

[B27] Li H. Durbin R. (2009). Fast and accurate short read alignment with burrows-wheeler transform. *Bioinformatics* 25 1754–1760. 10.1093/bioinformatics/btp324 19451168 PMC2705234

[B28] Li J. Besseau S. Toronen P. Sipari N. Kollist H. Holm L. (2013). Defense-related transcription factors WRKY70 and WRKY54 modulate osmotic stress tolerance by regulating stomatal aperture in *Arabidopsis*. *New Phytol.* 200 457–472. 10.1111/nph.12378 23815736 PMC4284015

[B29] Li J. Brader G. Kariola T. Palva E. T. (2006). WRKY70 modulates the selection of signaling pathways in plant defense. *Plant J.* 46 477–491. 10.1111/j.1365-313X.2006.02712.x 16623907

[B30] Liu P. Zhang S. Zhou B. Luo X. Zhou X. F. Cai B. (2019). The histone H3K4 demethylase JMJ16 represses leaf senescence in *Arabidopsis*. *Plant Cell* 31 430–443. 10.1105/tpc.18.00693 30712008 PMC6447021

[B31] Liu Y. Zhang A. Yin H. Meng Q. Yu X. Huang S. (2018). Trithorax-group proteins *Arabidopsis* Trithorax4 (ATX4) and ATX5 function in abscisic acid and dehydration stress responses. *New Phytol.* 217 1582–1597. 10.1111/nph.14933 29250818

[B32] Lu F. Cui X. Zhang S. Liu C. Cao X. (2010). JMJ14 is an H3K4 demethylase regulating flowering time in *Arabidopsis*. *Cell Res.* 20 387–390. 10.1038/cr.2010.27 20177424

[B33] Lu F. Li G. Cui X. Liu C. Wang X. J. Cao X. (2008). Comparative analysis of JmjC domain-containing proteins reveals the potential histone demethylases in *Arabidopsis* and rice. *J Integr. Plant Biol.* 50 886–896. 10.1111/j.1744-7909.2008.00692.x 18713399

[B34] Miura K. Renhu N. Suzaki T. (2020). The PHD finger of Arabidopsis SIZ1 recognizes trimethylated histone H3K4 mediating SIZ1 function and abiotic stress response. *Commun. Biol.* 3:23. 10.1038/s42003-019-0746-2 31925312 PMC6954211

[B35] Mosammaparast N. Shi Y. (2010). Reversal of histone methylation: Biochemical and molecular mechanisms of histone demethylases. *Annu. Rev. Biochem.* 79 155–179. 10.1146/annurev.biochem.78.070907.103946 20373914

[B36] Ng D. W. Wang T. Chandrasekharan M. B. Aramayo R. Kertbundit S. Hall T. C. (2007). Plant SET domain-containing proteins: Structure, function and regulation. *Biochim. Biophys. Acta* 1769 316–329. 10.1016/j.bbaexp.2007.04.003 17512990 PMC2794661

[B37] Rushton P. J. Somssich I. E. Ringler P. Shen Q. J. (2010). WRKY transcription factors. *Trends Plant Sci.* 15 247–258. 10.1016/j.tplants.2010.02.006 20304701

[B38] Santos-Rosa H. Schneider R. Bannister A. J. Sherriff J. Bernstein B. E. Emre N. C. (2002). Active genes are tri-methylated at K4 of histone H3. *Nature* 419 407–411. 10.1038/nature01080 12353038

[B39] Searle I. R. Pontes O. Melnyk C. W. Smith L. M. Baulcombe D. C. (2010). JMJ14, a JmjC domain protein, is required for RNA silencing and cell-to-cell movement of an RNA silencing signal in *Arabidopsis*. *Genes Dev.* 24 986–991. 10.1101/gad.579910 20478993 PMC2867213

[B40] Shen Y. Conde E. S. N. Audonnet L. Servet C. Wei W. Zhou D. X. (2014). Over-expression of histone H3K4 demethylase gene JMJ15 enhances salt tolerance in Arabidopsis. *Front. Plant Sci.* 5:290. 10.3389/fpls.2014.00290 25009544 PMC4068201

[B41] Shen Y. Devic M. Lepiniec L. Zhou D. X. (2015). Chromodomain, Helicase and DNA-binding CHD1 protein, CHR5, are involved in establishing active chromatin state of seed maturation genes. *Plant Biotechnol. J.* 13 811–820. 10.1111/pbi.12315 25581843

[B42] Shi Y. Lan F. Matson C. Mulligan P. Whetstine J. R. Cole P. A. (2004). Histone demethylation mediated by the nuclear amine oxidase homolog LSD1. *Cell* 119 941–953. 10.1016/j.cell.2004.12.012 15620353

[B43] Sokol A. Kwiatkowska A. Jerzmanowski A. Prymakowska-Bosak M. (2007). Up-regulation of stress-inducible genes in tobacco and *Arabidopsis* cells in response to abiotic stresses and ABA treatment correlates with dynamic changes in histone H3 and H4 modifications. *Planta* 227 245–254. 10.1007/s00425-007-0612-1 17721787

[B44] Song Q. Huang T. Y. Yu H. H. Ando A. Mas P. Ha M. (2019). Diurnal regulation of SDG2 and JMJ14 by circadian clock oscillators orchestrates histone modification rhythms in Arabidopsis. *Genome Biol.* 20:170. 10.1186/s13059-019-1777-1 31429787 PMC6892391

[B45] Sun Q. Zhou D. X. (2008). Rice JmjC domain-containing gene JMJ706 encodes H3K9 demethylase required for floral organ development. *Proc. Natl. Acad. Sci. U. S. A.* 105 13679–13684. 10.1073/pnas.0805901105 18765808 PMC2533249

[B46] Tsukada Y. Fang J. Erdjument-Bromage H. Warren M. E. Borchers C. H. Tempst P. (2006). Histone demethylation by a family of JmjC domain-containing proteins. *Nature* 439 811–816. 10.1038/nature04433 16362057

[B47] van Dijk K. Ding Y. Malkaram S. Riethoven J. J. Liu R. Yang J. (2010). Dynamic changes in genome-wide histone H3 lysine 4 methylation patterns in response to dehydration stress in *Arabidopsis thaliana*. *BMC Plant Biol.* 10:238. 10.1186/1471-2229-10-238 21050490 PMC3095321

[B48] Viana V. E. Busanello C. da Maia L. C. Pegoraro C. Costa de Oliveira A. (2018). Activation of rice WRKY transcription factors: An army of stress fighting soldiers? *Curr. Opin. Plant Biol.* 45 268–275. 10.1016/j.pbi.2018.07.007 30060992

[B49] Wang T. J. Huang S. Zhang A. Guo P. Liu Y. Xu C. (2021). JMJ17-WRKY40 and HY5-ABI5 modules regulate the expression of ABA-responsive genes in *Arabidopsis*. *New Phytol.* 230 567–584. 10.1111/nph.17177 33423315

[B50] Wang Y. Cui X. Yang B. Xu S. Wei X. Zhao P. (2020). WRKY55 transcription factor positively regulates leaf senescence and the defense response by modulating the transcription of genes implicated in the biosynthesis of reactive oxygen species and salicylic acid in *Arabidopsis*. *Development* 147:dev189647. 10.1242/dev.189647 32680933

[B51] Winter D. Vinegar B. Nahal H. Ammar R. Wilson G. V. Provart N. J. (2007). An “Electronic Fluorescent Pictograph” browser for exploring and analyzing large-scale biological data sets. *PLoS One* 2:e718. 10.1371/journal.pone.0000718 17684564 PMC1934936

[B52] Yang H. Han Z. Cao Y. Fan D. Li H. Mo H. (2012a). A companion cell-dominant and developmentally regulated H3K4 demethylase controls flowering time in Arabidopsis via the repression of FLC expression. *PLoS Genet.* 8:e1002664. 10.1371/journal.pgen.1002664 22536163 PMC3334889

[B53] Yang H. Mo H. Fan D. Cao Y. Cui S. Ma L. (2012b). Overexpression of a histone H3K4 demethylase, JMJ15, accelerates flowering time in *Arabidopsis*. *Plant Cell Rep.* 31 1297–1308. 10.1007/s00299-012-1249-5 22555401

[B54] Yang R. Hong Y. Ren Z. Tang K. Zhang H. Zhu J. K. (2019). A role for pickle in the regulation of cold and salt stress tolerance in *Arabidopsis*. *Front. Plant Sci.* 10:900. 10.3389/fpls.2019.00900 31354770 PMC6633207

[B55] Yang W. Jiang D. Jiang J. He Y. (2010). A plant-specific histone H3 lysine 4 demethylase represses the floral transition in *Arabidopsis*. *Plant J.* 62 663–673. 10.1111/j.1365-313X.2010.04182.x 20202164

[B56] Yang Z. Qiu Q. Chen W. Jia B. Chen X. Hu H. (2018). Structure of the *Arabidopsis* JMJ14-H3K4me3 complex provides insight into the substrate specificity of KDM5 subfamily histone demethylases. *Plant Cell* 30 167–177. 10.1105/tpc.17.00666 29233856 PMC5810570

[B57] Zhang X. Bernatavichute Y. V. Cokus S. Pellegrini M. Jacobsen S. E. (2009). Genome-wide analysis of mono-, di- and trimethylation of histone H3 lysine 4 in *Arabidopsis thaliana*. *Genome Biol.* 10:R62. 10.1186/gb-2009-10-6-r62 19508735 PMC2718496

[B58] Zheng D. Wang L. Chen L. Pan X. Lin K. Fang Y. (2019). Salt-responsive genes are differentially regulated at the chromatin levels between seedlings and roots in rice. *Plant Cell Physiol.* 60 1790–1803. 10.1093/pcp/pcz095 31111914

[B59] Zheng Y. Ding Y. Sun X. Xie S. Wang D. Liu X. (2016). Histone deacetylase HDA9 negatively regulates salt and drought stress responsiveness in Arabidopsis. *J. Exp. Bot.* 67 1703–1713. 10.1093/jxb/erv562 26733691

[B60] Zong W. Zhong X. You J. Xiong L. (2013). Genome-wide profiling of histone H3K4-tri-methylation and gene expression in rice under drought stress. *Plant Mol. Biol.* 81 175–188. 10.1007/s11103-012-9990-2 23192746

