# Supplementary figures and images for: Involvement of JMJ15 in the dynamic change of genome-wide H3K4me3 in response to salt stress
Source: Front Plant Sci. 2022 Sep 26;13:1009723. doi: 10.3389/fpls.2022.1009723 (PMC9549339; doi:10.3389/fpls.2022.1009723)

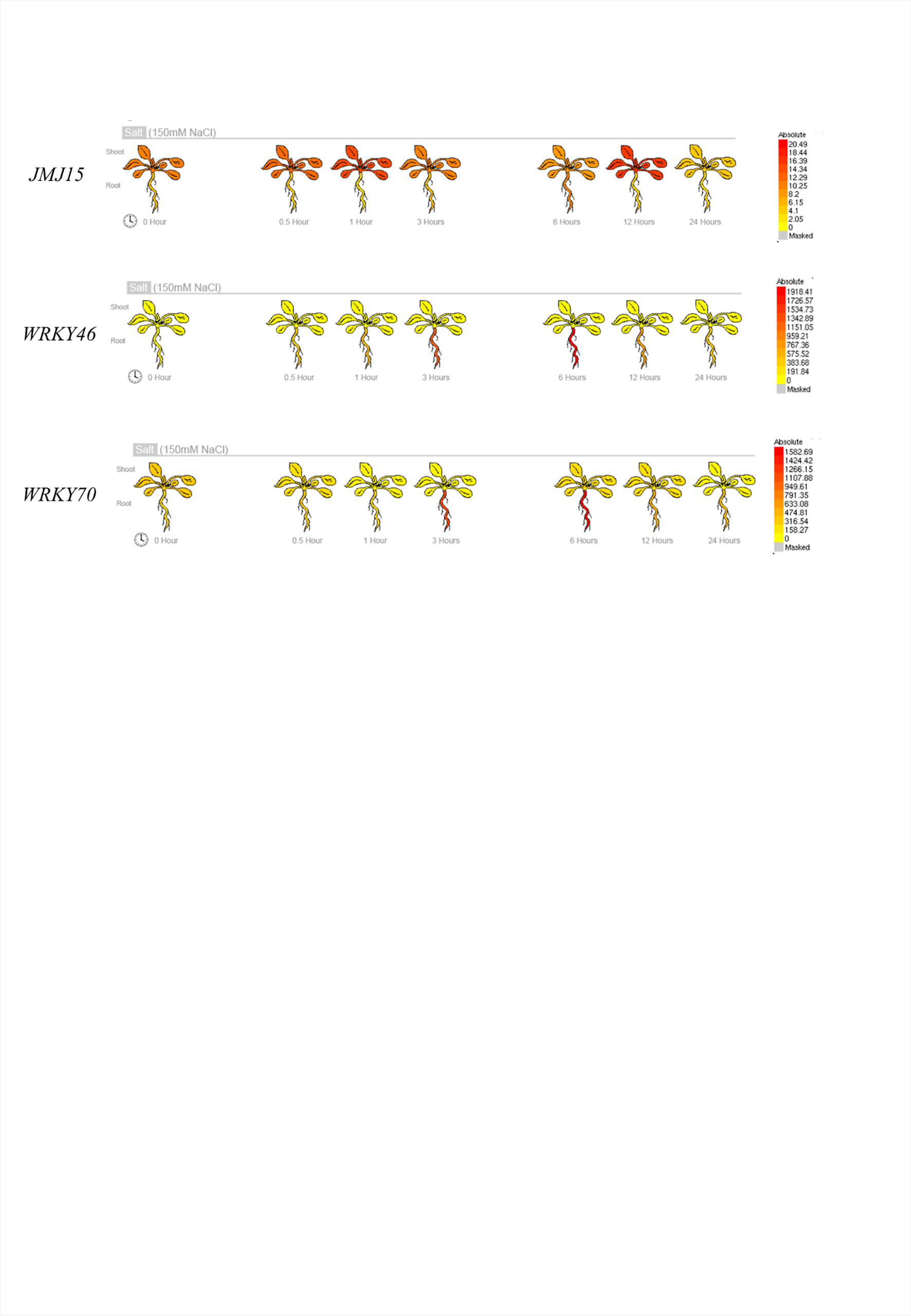

Supplement: Supplementary Figure 1 — Expression patterns of JMJ15, WRKY46, and WRKY70 under 150 mM NaCl treatment for 24 h. The expression data were obtained from a public database, eFP browser (http://bar.utoronto.ca/efp_arabidopsis/cgi-bin/efpWeb.cgi). The color bar on the right indicates the absolute expression values. [file Image_1.TIFF]
